# Detection of pTDP‐43 via routine muscle biopsy: A promising diagnostic biomarker for amyotrophic lateral sclerosis

**DOI:** 10.1111/bpa.13261

**Published:** 2024-04-11

**Authors:** Qi‐Jie Zhang, Jie Lin, You‐Liang Wang, Long Chen, Ying Ding, Fu‐Ze Zheng, Huan‐Huan Song, Ao‐Wei Lv, Yu‐Ying Li, Qi‐Fu Guo, Min‐Ting Lin, Wei Hu, Liu‐Qing Xu, Wen‐Long Zhao, Ling Fang, Meng‐Chao Cui, Zhi‐Fei Fu, Wan‐Jin Chen, Jing Zhang, Zhi‐Qiang Wang, Ning Wang, Ying Fu

**Affiliations:** ^1^ Department of Neurology, Fujian Institute of Neurology, The First Affiliated Hospital Fujian Medical University Fuzhou China; ^2^ Department of Neurology, National Regional Medical Center, Binhai Campus of The First Affiliated Hospital Fujian Medical University Fuzhou China; ^3^ Fujian Key Laboratory of Molecular Neurology Fujian Medical University Fuzhou China; ^4^ Key Laboratory of Radiopharmaceuticals, Ministry of Education, College of Chemistry Beijing Normal University Beijing China; ^5^ Public Technology Service Center Fujian Medical University Fuzhou China; ^6^ Department of Pathology, The First Affiliated Hospital Zhejiang University School of Medicine Hangzhou China; ^7^ National Human Brain Bank for Health and Disease Zhejiang University Hangzhou China

**Keywords:** amyotrophic lateral sclerosis, diagnostic marker, muscle biopsy, pathology, phosphorylated TDP‐43

## Abstract

Amyotrophic lateral sclerosis (ALS) is a devastating neurodegenerative disease, pathologically characterized by TDP‐43 aggregates. Recent evidence has been indicated that phosphorylated TDP‐43 (pTDP‐43) is present not only in motor neurons but also in muscle tissues. However, it is unclear whether testing pTDP‐43 aggregation in muscle tissue would assist in the diagnosis of ALS. We propose three key questions: (i) Is aggregation of pTDP‐43 detectable in routine biopsied muscles? (ii) Can detection of pTDP‐43 aggregation discriminate between ALS and non‐ALS patients? (iii) Can pTDP‐43 aggregation be observed in the early stages of ALS? We conducted a diagnostic study comprising 2 groups: an ALS group in which 18 cases underwent muscle biopsy screened from a registered ALS cohort consisting of 802 patients and a non‐ALS control group, in which we randomly selected 54 muscle samples from a biospecimen bank of 684 patients. Among the 18 ALS patients, 3 patients carried pathological GGGGCC repeats in the C9ORF72 gene, 2 patients carried SOD1 mutations, and 7 patients were at an early stage with only one body region clinically affected. The pTDP‐43 accumulation could be detected in routine biopsied muscles, including biceps brachii, deltoid, tibialis anterior, and quadriceps. Abnormal aggregation of pTDP‐43 was present in 94.4% of ALS patients (17/18) compared to 29.6% of non‐ALS controls (16/54; *p* < 0.001). The pTDP‐43 aggregates were mainly close to the sarcolemma. Using a semi‐quantified pTDP‐43 aggregates score, we applied a cut‐off value of 3 as a diagnostic biomarker, resulting in a sensitivity of 94.4% and a specificity of 83.3%. Moreover, we observed that accumulation of pTDP‐43 occurred in muscle tissues prior to clinical symptoms and electromyographic lesions. Our study provides proof‐of‐concept for the detection of pTDP‐43 accumulation via routine muscle biopsy which may serve as a novel biomarker for diagnosis of ALS.

## Graphical abstract

To explore whether testing pTDP‐43 aggregation in muscle tissue would assist in the diagnosis of ALS, we conducted a diagnostic study in 18 ALS patients and 54 randomly matched controls from ALS and muscular disease cohorts. Our results indicated that pTDP‐43 was more prone to accumulation in ALS patients, enabling differentiation between ALS and non‐ALS patients with high sensitivity and specificity. Moreover, we observed that accumulation of pTDP‐43 occurred in muscle tissues prior to clinical symptoms and electromyographic lesions.

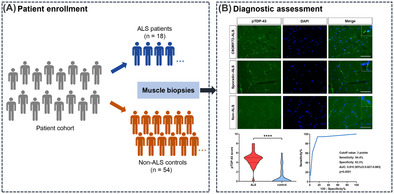

## 1 INTRODUCTION

Amyotrophic lateral sclerosis (ALS), also known as Lou Gehrig's disease, is the most common and universally fatal motor neuron degenerative disease [1]. Symptoms often begin with adult‐onset limb weakness, progressing to the trunk and bulbar muscles, ultimately leading to respiratory failure within 3–5 years of onset [2]. Hitherto, the diagnosis of ALS remains clinical, predominantly based upon progressive motor impairment, electrophysiological testing, and gene mutation screening [3]. However, because of the diverse clinical manifestations and lag in electrophysiological characterization, as well as the high incidence of sporadic ALS, previous studies have shown a high misdiagnosis rate (up to 10%) in ALS [4, 5], and a median diagnosis delay of approximately 14 months [6]. Therefore, a biomarker for early and accurate diagnosis of ALS is urgently required.

The core pathological characteristics of ALS include abnormal protein deposits in motor neurons and glial cells [7], and diverse aggregation‐prone proteins have been previously identified, including TAR DNA‐binding protein 43 kDA (TDP‐43), fused‐in‐sarcoma protein (FUS), superoxide dismutase 1 (SOD1), Optineurin (OPTN), Ubiquilin‐2 (UBQLN2), Ataxin‐2 (ATXN2), and p62 [8]. Among them, phosphorylated TDP‐43 (pTDP‐43) which has been localized incorrectly to the cytoplasm, is a key pathological feature. Through postmortem muscle tissue examination of ALS, a previous study demonstrated that pTDP‐43 aggregates in the axial skeletal muscle of 19 out of 57 patients (33.3%) [9]; however, another study demonstrated that pTDP‐43 aggregates in the skeletal muscle of 28 out of 30 patients (93.3%) [10]. Interestingly, a retrospective cohort study of ALS biopsy muscle tissue revealed that pTDP‐43 aggregated in nerve bundles of skeletal muscles in 33 out of 36 patients (91.7%) [11]. All of the above studies suggest that there is a relationship between pTDP‐43 aggregation within muscle tissue and ALS. However, previous studies mostly used autopsy muscle samples. It is therefore necessary to find out whether testing pTDP‐43 aggregation in a routine biopsy of skeletal muscle would help in the diagnosis of ALS.

To assess the sensitivity and specificity of pTDP‐43 aggregation in muscle of ALS patients through biopsy muscle‐tissue pathology examination, we propose three key facets to our research: (i) Is aggregation of pTDP‐43 detectable in routine biopsied muscles and does it vary across different biopsy sites? (ii) Can detection of pTDP‐43 aggregation discriminate between ALS and non‐ALS patients? (iii) Can pTDP‐43 aggregation be observed in the early stages of ALS? To answer these questions, we conducted a diagnostic study to determine the pathological characteristics of pTDP‐43 aggregation in routine muscle biopsy‐derived samples from a registered ALS cohort, with a special focus on early‐stage patients (those with single segment involvement or atypical electromyographic abnormalities). Notably, we enrolled a control group of non‐ALS patients with confirmed genetic or pathological diagnosis, which covered a wide range of muscular diseases containing previously reported TDP‐43 aggregates. Our study, based on the strengths of genetic and pathological diagnosis, aims to provide compelling evidence for the diagnostic value of pTDP‐43 aggregates in biopsied muscle tissue for ALS.

## 2 MATERIALS AND METHODS

### 2.1 Study design and participants

**FIGURE 1:**
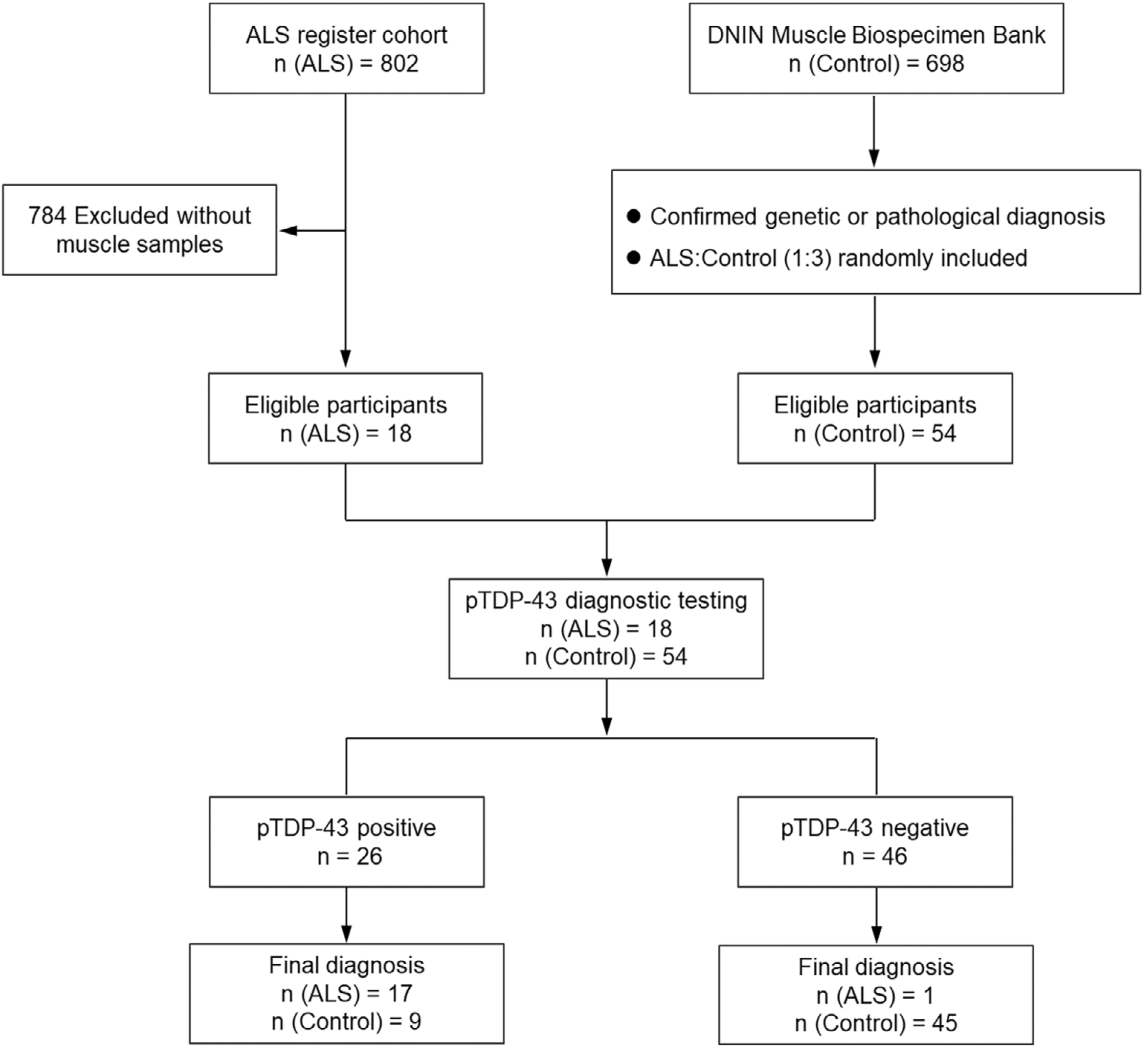
The patient flow chart for this study. After ROC analysis, pTDP‐43 score ≥3 was defined as positive.

This was a diagnostic study conducted by Department of Neurology and Institute of Neurology (DNIN) of the First Affiliated Hospital, Fujian Medical University between September 2022 and February 2023, the design and flow diagram of which are illustrated in Figure 1.

### 2.2 ALS patients

We recruited ALS patients from a registered ALS cohort from DNIN (NCT04008329). Based on this registered ALS cohort, we have reported several ALS prognostic factors previously [12, 13, 14]. All patients were diagnosed by two experienced neurologists (Q.J.Z. and N.W.) according to the Airlie House ALS diagnostic criteria [15]. Clinical assessments were performed by one trained team using a standardized inventory, and the neurological examinations were performed in a Neurological Evaluation Room to ensure standardization and the reliability of data. Baseline demographic information and detailed clinical profiles were collected, including age at onset, site of onset, history of disease progression, behavior change or dementia, family history, physical examination, neuroimaging, electrophysiological information and laboratory tests, such as routine blood tests, serum creatine kinase (CK) levels, serum vitamin 12 levels, hepatic function, renal function, and so on. The interval of follow‐up was three to 6 months and was conducted by the same neurologists (Q.J.Z. and N.W.).

Age at onset was defined as age at first symptom onset. Site of onset was defined as the site of first symptom onset, including bulbar, upper limb, or lower limb onset. ALS staging was defined based on the King's College staging system [16]. Positive family history was defined as at least two ALS patients in the same family. We also established a genetic screening system in our center. We detected GGGGCC repeats in the *C9ORF72* gene using repeat‐primed polymerase chain reaction as described previously [17], and whole‐exome sequencing (WES) was conducted on *C9ORF72*‐negative ALS patients to identify other gene variants, using a Hiseq2500 platform (Illumina, San Diego, CA), as described [18]. The identified candidate disease‐causing mutations were further verified by Sanger sequencing (*SOD1* c.214C > T; *SOD1* c.449 T > C; *FUS* c. 1574C > T).

Out of a total of 802 registered ALS patients, 18 cases underwent muscle biopsy for the purpose of diagnosis. The common indications for muscle biopsy referral were a lower motor neuron syndrome, early disease stage limited to one body region, without confirmed neuropathic lesions (even showing myopathic changes) in EMG studies.

### 2.3 Non‐ALS controls

These controls were serially recruited from the neuromuscular cohort of DNIN. The non‐ALS patients were diagnosed by two experienced neurologists, Prof. Z.Q.W. and Prof. N.W. As this center specializes in pathology and genetics, it attracts patients with neuromuscular diseases from across the country, and we have therefore reported nationwide epidemiological data and identified new causative genes for diverse neuromuscular diseases [19, 20, 21].

Routine muscle biopsies were processed according to standardized protocols, and biopsied muscles were frozen in liquid nitrogen‐cooled isopentane in the Muscle Biospecimen Bank of this center. Between January 2010 and December 2022, a total of 698 muscle biopsies were performed in this cohort. In this study, we selected 54 muscle samples, with the principles for control selection including: (i) patients with a confirmed genetic or pathological assessment and (ii) random selection.

### 2.4 TDP‐43/pTDP‐43 immunofluorescence staining

Frozen muscle biopsies were cryo‐sectioned at a thickness of 7 μm, mounted onto slides, and air dried for 20 min at room temperature. For immunofluorescence labeling, sections were washed with PBS, fixed in 4% PFA for 20 min, permeabilized with 0.1% Triton, and blocked with normal donkey serum (Jackson ImmunoResearch). Thereafter, the sections were incubated with primary rabbit antibodies including TDP‐43 (C‐terminal) polyclonal antibody (1:500; 12,892‐1‐AP, Proteintech), and phospho‐TDP43 (Ser409/410) recombinant antibody (1:500; 80,007‐1‐RR, Proteintech) overnight at 4°C. After washing 3 × 5 min with PBS, sections were incubated with secondary antibodies at room temperature for 1 h (1:300, donkey anti‐rabbit IgG (H + L) conjugated with Alexa Fluor 488, Jackson ImmunoResearch). Cover slips were applied after counterstaining the nucleus with 4′,6‐diamidino‐2‐phenylindole, and sections were visualized using an EVOS M5000 fluorescence microscope (Invitrogen).

### 2.5 pTDP‐43 immunohistochemical assay

After fixation, skeletal muscles were embedded in paraffin blocks, cut into 6 μm sections and mounted on glass slides. Then, following immersion in xylene, hydration through graded ethanol solutions, heat‐mediated antigen retrieval and elimination of endogenous peroxidase activity by incubation in hydrogen peroxide. Subsequently, the sections were incubated with phospho‐TDP43 (Ser409/410) recombinant antibody (1:500; 80,007‐1‐RR, Proteintech). The next day, sections were incubated with secondary antibody, developed with 3,3′‐diaminobenzidine (DAB) and counterstained with Harris Hematoxylin. Light microscopy was used for identification of sections using BX53 DIX/BF Olympus research microscope with an attached DP27 digital camera (Olympus, America).

### 2.6 Aggregates conformation‐specific probe fluorescent staining

To specifically detect the spatial conformation of TDP43 aggregates, we used a fluorescent probe capable of recognizing the β‐fold structure for fluorescent staining, as previous reported [22]. In short, the slices were incubated in aqueous solutions of DANIR 8c (1 μM) for 10 min at room temperature and then washed with 60% ethanol. Sections were observed using Zeiss 7800 confocal microscope. To determine the differences of TDP‐43 phosphorylation sites and its spatial conformations, we used phospho‐TDP43 (Ser409/410) antibody and DANIR 8c to observe the colocalization through super‐resolution microscopy (Multi‐SIM, Naxi‐Tech).

### 2.7 Electron microscopy

The biopsy muscle samples were fixed with 2.5% glutaraldehyde, post fixed in buffered 2% osmium tetroxide, dehydrated in a graded alcohol series, and embedded in epoxy resin. 70‐nm ultrathin sections were collected using a LEICA EM UC7 (Leica Microsystems, Buffalo Grove, Israel), stained with uranyl acetate and lead citrate, and visualized with FEI Tecnai G2 transmission electron microscope (Hillsboro, America).

### 2.8 pTDP‐43 pathological semi‐quantitative evaluation

The scoring method can influence the result when examining this many muscle samples. A previous study demonstrated that pTDP‐43 aggregates in the skeletal muscles of 93.3% of patients with a semi‐quantitative evaluation based upon five muscle regions (tongue, cervical muscle, diaphragm, iliopsoas muscle, and heart) [10]. In this study, we also employed this semi‐quantitative evaluation based on single muscle region.

The rules of this semi‐quantitative evaluation were (1) at least three different fields were photographed randomly in every patient and (2) we counted pTDP‐43 positive cells in each field and calculated the mean proportion of pTDP‐43 positive cells (pTDP‐43 Positive cell%). The pTDP‐43 score was then defined as a range from 0 to 10 with the 10% interval of pTDP‐43 positive cells, 0 = “all negative cells”, 10 = “90%<pTDP‐43 Positive cell% <100%”.

The samples were randomly coded and blinded, and the results were then interpreted by two people. If the interpretation results were inconsistent, a third person interpreted the results. There was a high inter‐reader agreement (Cohen's Kappa: 0.938, 95% CI: 0.854–1.022).

### 2.9 Statistical analysis

The sample size for diagnostic tests was calculated according to the following formula:

$$n={\left(\frac{Z_{\alpha }}{\delta }\right)}^{2}\left(1-P\right)P.$$

where *n* is the required sample size. *Zα* is the *z* value when the cumulative probability equals α over 2 in a normal distribution, and when α is 0.05, *Zα* is 1.96. δ is the allowable error, which is often equal to 0.1. *P* is the sensitivity or specificity. The expected sensitivity was 95% and the specificity was 85%. The sample size required for sensitivity was estimated (sample size of the ALS group) to be 18. The sample size required for the specificity (the sample size of control group) was calculated to be 49. As the control group was set at three times the number of patients in ALS group, the higher number, 54 was used.

A Kolmogorov–Smirnov z test was used to examine the distribution type. The mean ± standard deviation was used to represent the measurement data conforming to a normal distribution, and the median was used to represent the measurement data not conforming to a normal distribution. The diagnostic sensitivity and specificity were calculated. The statistical analysis was carried out using SPSS v.19.0 software.

## 3 RESULTS

### 3.1 Clinical characteristics and dataset overview

Among the 18 ALS patients, only two patients (ID: ALS‐13 and ALS‐15) had a positive family history of ALS, with the remainder being sporadic cases. After genetic analysis, three patients (ID: ALS‐06, ALS‐09, and ALS‐15) carried pathological GGGGCC repeats in *C9ORF72* gene, two patients (ID: ALS‐05 and ALS‐13) carried *SOD1* mutations, and one patient (ID: ALS‐07) had a *FUS* mutation. Detailed demographic and clinical characteristics of all ALS patients are listed in Table 1.

**TABLE 1:** The demographic and clinical characteristics of ALS patients.

Patient ID	Gender	Family history	AAO (y)	SOO (1 = Bul,2 = UL, 3 = LL)	Clinical phenotype	Clinically affected regions when muscle biopsy	EMG testing when muscle biopsy	Muscle biopsy					King's college stage when muscle biopsy	Final diagnosis (based on Airlie diagnostic criteria)	Gene mutation screening	Survival (m)
								Duration until muscle biopsy (m)	Site of muscle biopsy (1 = biceps, 2 = deltoid, 3 = quadriceps, 4 = tibialis anterior)	Pathological diagnosis	Histopathology grading scale (cumulative score; range from 0 to 12 points)	pTDP‐43 score				
ALS‐01	Male	No	50	2	LMN + UMN	3	FPs + sw; without confirmed neurogenic lesions	53	1	Neurogenic	4	4	4B	Definite ALS	*C9ORF72* (−), *ATXN2* (−), WES (−)	60
ALS‐02	Female	No	71	3	LMN + UMN	2	Myogenic lesions	15	1	Neurogenic	3	5	2B	Probable ALS	ND	31
ALS‐03	Female	No	62	1	UMN‐dominant	3	Myogenic lesions	36	1	Neurogenic	3	4	3	Definite ALS	*C9ORF72* (−), *ATXN2* (−), WES (−)	57
ALS‐04	Female	No	52	3	LMN‐dominant	2	Neugenic lesions	4	1	Neurogenic	1	5	2B	Possible ALS	*C9ORF72* (−), *ATXN2* (−), WES (−)	12
ALS‐05	Female	No	51	2	LMN‐dominant	2	Myogenic lesions	5	1	Neurogenic	3	7	2B	Definite ALS (genetic)	*SOD1*: c.214C > T, p.H72Y	12
ALS‐06	Female	No	54	1	LMN + UMN	3	Neugenic lesions	70	2	Neurogenic	2	8	3	Definite ALS (genetic)	*C9ORF72*(GGGGCC): *n* > 90	Alive (>79)
ALS‐07	Female	No	13	3	UMN‐dominant	3	Neugenic lesions	23	2	Neurogenic	2	0	3	Definite ALS (genetic)	*FUS*: c.1574C > T, p.P525L	Alive (>111)
ALS‐08	Female	No	46	2	LMN‐dominant	2	Neugenic lesions	26	4	Neurogenic	3	5	2B	Possible ALS	*C9ORF72* (−), *ATXN2* (−), WES (−)	Alive (>39)
ALS‐09	Male	No	55	1	LMN + UMN	1	Neugenic lesions	16	1	Neurogenic	1	5	1	Definite ALS (genetic)	*C9ORF72*(GGGGCC): *n* > 86	Alive (>28)
ALS‐10	Male	No	73	1	LMN + UMN	1	FPs + sw; without confirmed neurogenic lesions	17	1	Neurogenic	1	6	1	Probable ALS	*C9ORF72* (−), *ATXN2* (−), WES (−)	Alive (>29)
ALS‐11	Male	No	48	2	LMN + UMN	2	Neugenic lesions	8	1	neurogenic	2	5	2B	Probable ALS	*C9ORF72* (−), *ATXN2* (−), WES (−)	19
ALS‐12	Male	No	44	1	LMN + UMN	1	Neugenic lesions	9	2	Neurogenic	1	5	1	Possible ALS	*C9ORF72* (−), *ATXN2* (−), WES (−)	Alive (>18)
ALS‐13	Female	Yes	35	2	LMN‐dominant	1	Neugenic lesions	6	1	Neurogenic	2	3	1	Definite ALS (genetic)	*SOD1*: c.449 T > C, p.I150T	Alive (>13)
ALS‐14	Male	No	72	3	LMN + UMN	2	Neugenic lesions	20	3	Neurogenic	1	5	2B	Probable ALS	*C9ORF72* (−), *ATXN2* (−), WES (−)	Alive (>39)
ALS‐15	Male	Yes	47	1	LMN + UMN, dementia	1	FPs + sw; without confirmed neurogenic lesions	19	1	Neurogenic	1	4	1	Definite ALS (genetic)	*C9ORF72*(GGGGCC): *n* > 60	Alive (>25)
ALS‐16	Female	No	62	2	LMN + UMN	1	Neugenic lesions	8	1	Neurogenic	1	5	1	Possible ALS	*C9ORF72* (−), *ATXN2* (−), WES (−)	Alive (>14)
ALS‐17	Female	No	56	2	LMN + UMN	2	Neugenic lesions	13	1	Neurogenic	1	4	2B	Probable ALS	*C9ORF72* (−), *ATXN2* (−), WES (−)	Alive (>18)
ALS‐18	Female	No	45	2	LMN + UMN	1	Neugenic lesions	5	1	Neurogenic	1	3	1	Possible ALS	*C9ORF72* (−), *ATXN2* (−), WES (−)	Alive (>7)

Abbreviations: AAO, age at onset; Bul, bulbar; EMG, electromyography; FPs, fibrillation potentials; LL, lower limb; LMN, lower motor neuron; ND, not detected; SOO, site of onset; sw, positive sharp waves; UL, upper limb; UMN, upper motor neuron; WES, whole‐exome sequencing.

Among the non‐ALS patients, there were 32 males and 22 females. Aiming to clarify TDP‐43/pTDP‐43 pathological features, we enrolled patients who had diverse neurological diseases, including facioscapulohumeral muscular dystrophy (FSHD; *n* = 10), limb girdle muscular dystrophy (LGMD; *n* = 8), inflammatory myopathy (*n* = 8), lipid storage myopathy (LSM; *n* = 5), mitochondrial disease (*n* = 5), glycogen storage disease (GSD; *n* = 3), oculopharyngeal muscular dystrophy (OPMD; *n* = 3), oculopharyngodistal myopathy (OPDM; *n* = 2), myotonic dystrophy (*n* = 2), progressive muscular dystrophy (*n* = 2), periodic paralysis (*n* = 1), Charcot–Marie‐Tooth (CMT; *n* = 1), neuronal intranuclear inclusion disease (NIID; *n* = 1), inclusion body myositis (IBM; *n* = 1), McLeod syndrome (*n* = 1), and psychogenic disorder (*n* = 1). In non‐ALS patients, diagnosis was based on either genetic or muscle pathological evidence. The detailed demographic and clinical characteristics of non‐ALS patients are listed in Table S1.

### 3.2 pTDP‐43 pathological aggregation in routine muscular biopsies

**FIGURE 2:**
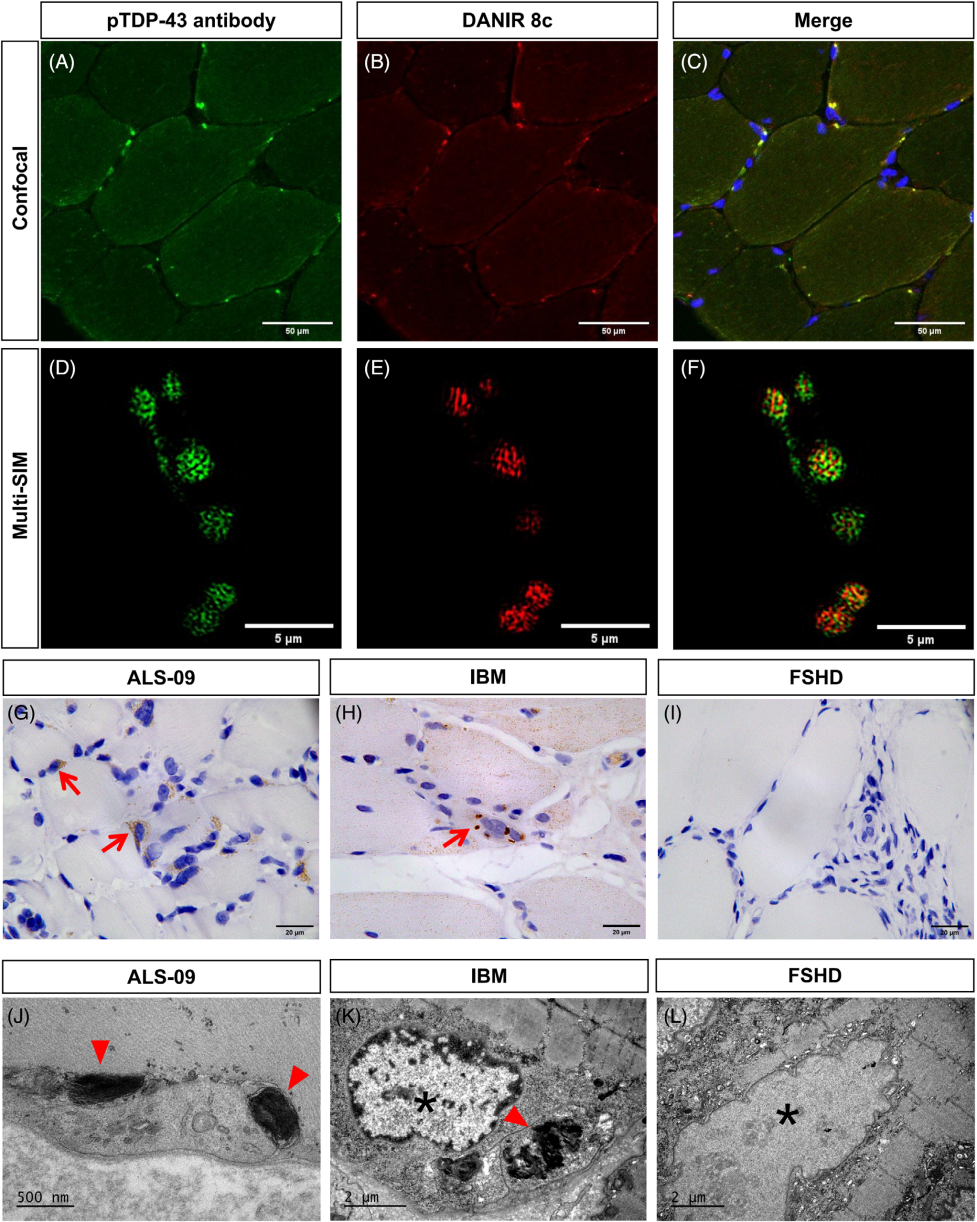
Fluorescence staining of TDP‐43 phosphorylation and its conformation by super‐resolution microscopy, immunohistochemical assay, and electron microscopy. (A–C) Immunofluorescence showed significant co‐localization of pTDP‐43 antibody and DANIR 8c probe, labeling aberrantly aggregated pTDP‐43 protein using confocal microscopy. (D–F) Immunofluorescence of pTDP‐43 antibody and DANIR 8c probe using super‐resolution microscopy (Multi‐SIM). (G–I) Immunohistochemical assay showed pTDP‐43 aggregates in muscle samples from ALS and IBM patients, while not in FSHD. (J–L) Electron microscopy showed phathological aggregations in muscle samples from ALS and IBM patients, while not in FSHD. The aggregates were mainly localized in cytoplasm and perinuclear regions of myofibers (red triangle). *Indicated the nuclei of myofiber.

All ALS and non‐ALS patients exhibited expression of TDP‐43, which was mainly localized to the nuclei of myofibers (Figure S1). Through muscle tissue fluorescent staining using pTDP‐43 antibody, we found significant abnormal aggregation of pTDP‐43 in ALS patients under confocal and super‐resolution microscopy, and the pTDP‐43 aggregates were mainly close to the sarcolemma (Figure 2A,D). We further confirmed the β‐fold structure of aggregated proteins using DANIR 8c probe (Figure 2B,C,E,F). Pathological aggregation was also confirmed by immunohistochemistry of pTDP‐43 (Figure 2G,I) and electron microscopy (Figure 2J,L), which showed that the aggregates were mainly localized in cytoplasm and perinuclei.

**FIGURE 3:**
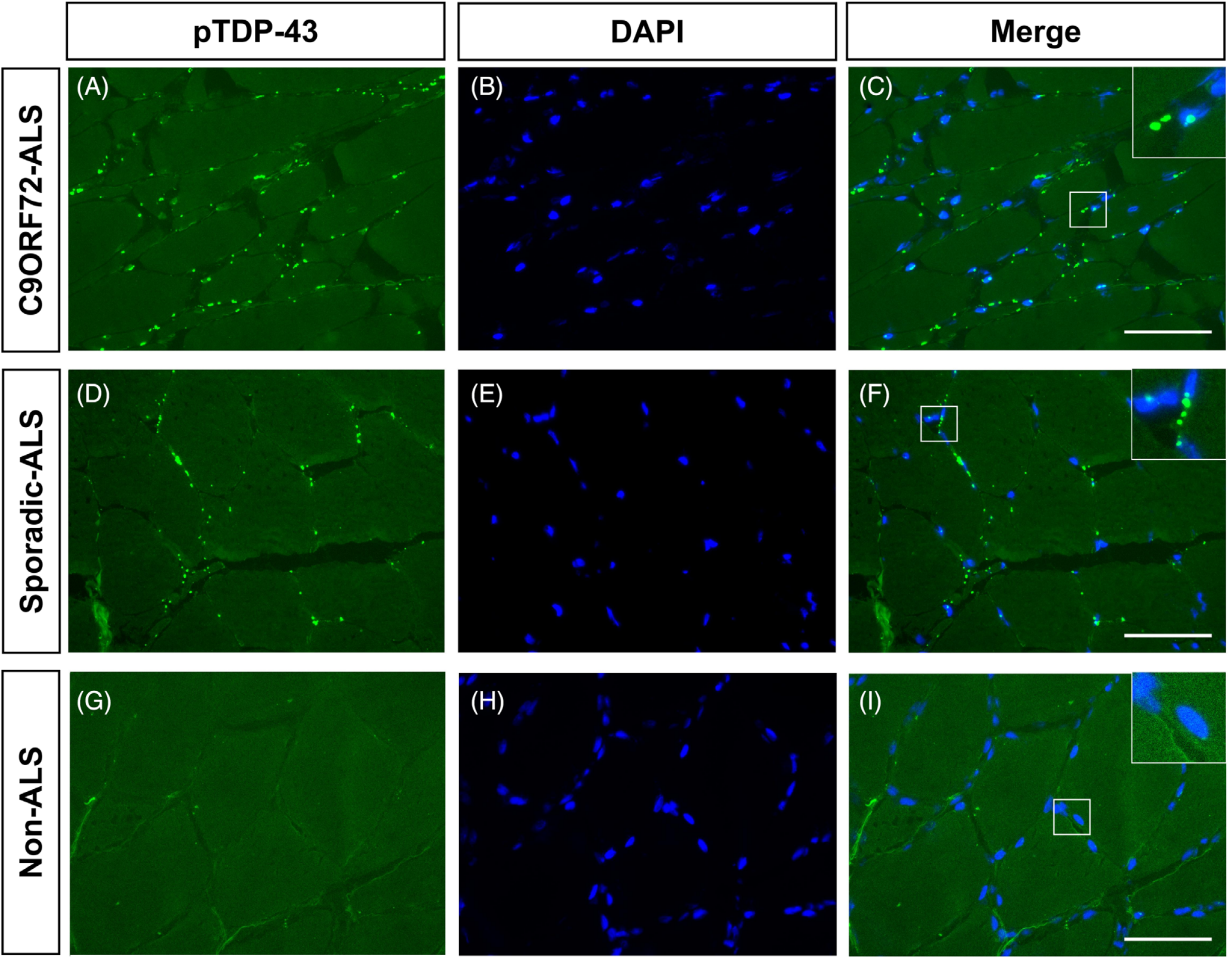
Immunofluorescence stained with anti‐pTDP‐43 in patients with *C9ORF72*‐ALS, sporadic‐ALS, and non‐ALS patient. (A–C) A remarkable amount of pTDP‐43 aggregates in myofibers from a ALS patient with *C9ORF72* (ID: ALS‐06). (D–F) Obvious pTDP‐43 accumulation in a sporadic‐ALS patient (ID: ALS‐11). (G–I) No pTDP‐43 deposit in non‐ALS patient, a lipid storage myopathy (LSM) with ETFDH gene mutation (ID: Ctrl‐25). Scale bar, 75 μm.

**FIGURE 4:**
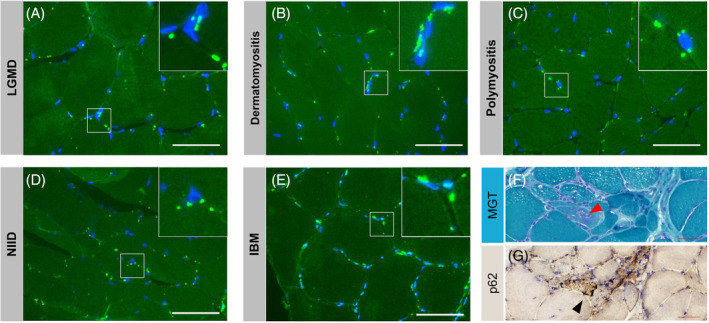
Immunofluorescence stained with anti‐pTDP‐43 in patients with non‐ALS. Obvious pTDP‐43 accumulation was observed in a LGMD patient (ID: Ctrl‐01; A), a dermatomyositis (Ctrl‐14; B), a polymyositis patient (ID: Ctrl‐15; C), a NIID patient (ID: Ctrl‐41; D), and an IBM patient (ID: Ctrl‐53; E). (F) The IBM patient (Ctrl‐53) showed rimmed vacuoles (red arrow) stained with MGT. (G) The rimmed vacuoles were p62 positive (black arrow). Scale bar, 75 μm (A–E); 50 μm (F–G).

In total, abnormal aggregation of pTDP‐43 was present in 17 of 18 ALS patients (94.4%) as opposed to 16 of 54 non‐ALS controls (29.6%, *p* < 0.001). Among the ALS muscle biopsies, abnormal aggregation of pTDP‐43 was detected across different routine biopsy sites, including biceps brachii (13/13), deltoid (2/3), tibialis anterior (1/1), and quadriceps (1/1). Representative images of pTDP‐43 pathology are shown in Figure 3. Notably, pTDP‐43 identification was negative in the muscles of a juvenile‐onset ALS patient carrying a *FUS* P525L mutation. (Detailed muscle pathology was shown in Figure S2) Furthermore, we observed obvious pTDP‐43 aggregations in LGMD, IM, NIID, and IBM (Figure 4).

### 3.3 pTDP‐43 pathology: A valuable tissue marker for ALS

**FIGURE 5:**
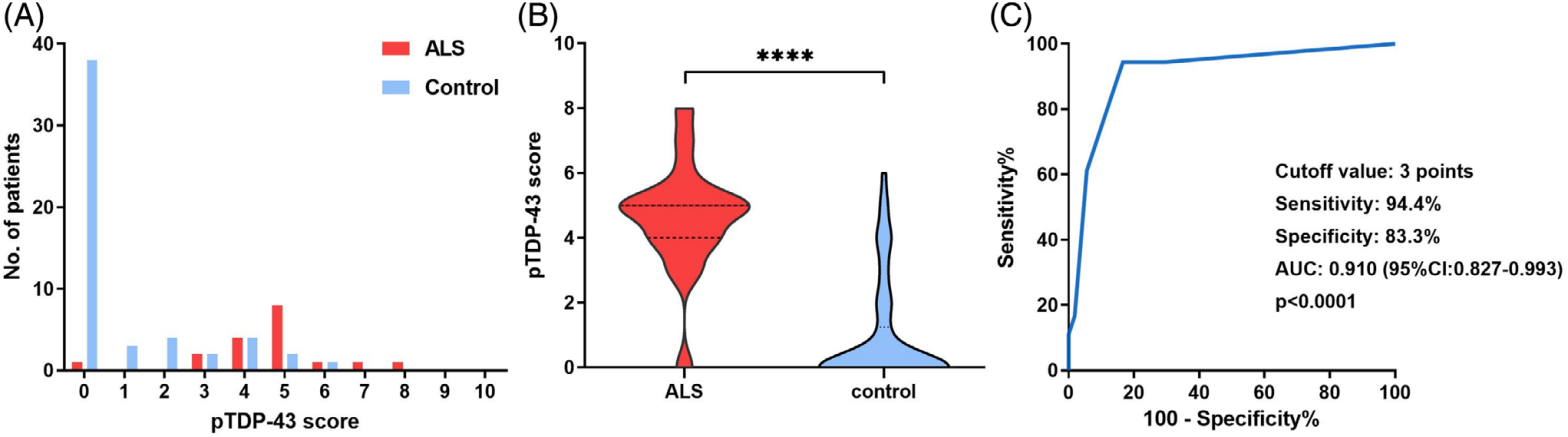
The diagnostic value of pTDP‐43 score. (A) Distribution of pTDP‐43 score in ALS patients and non‐ALS controls. (B) Comparison of pTDP‐43 score between ALS patients and non‐ALS controls. (C) The sensitivity and specificity of pTDP‐43 score in distinguishing ALS from controls analyzing by ROC.

To quantify of pTDP‐43 protein aggregation, we employed a visually dependent semi‐quantitative score (pTDP‐43 score) to assess pTDP‐43 inclusion density (Figure S3). From the pTDP‐43 score distribution histogram (Figure 5A), it was noted that ALS patients were all concentrated around 5 points, while non‐ALS patients were distributed at 0 points. A significant difference was found in pTDP‐43 score among ALS patients and non‐ALS controls (4.7 ± 0.4 vs. 0.90 ± 0.2, *p* < 0.001; Figure 5B). To assess the sensitivity and specificity of the pTDP‐43 score in distinguishing between ALS patients and non‐ALS controls, we performed ROC curve analysis, which yielded a pTDP‐43 score cut‐off value of 3 points with an area under the curve of 0.910 (95% CI: 0.827–0.993). Therefore, we determined pTDP‐43 score ≥3 as positive and it could be used as a diagnostic biomarker, which resulted in 94.4% sensitivity, 83.3% specificity (Figures 1, 5).

### 3.4 pTDP‐43 aggregation: An early event in ALS

We detected pTDP‐43 aggregation in the early stage of ALS patients. At the time of muscle biopsy, seven early‐stage ALS patients had only one body region affected clinically (Figure S4). Upon EMG, three patients (ID: ALS‐02, ALS‐03, ALS‐05) had myopathic damage at baseline, and during follow‐up, typical neuropathic lesions were presented gradually.

**FIGURE 6:**
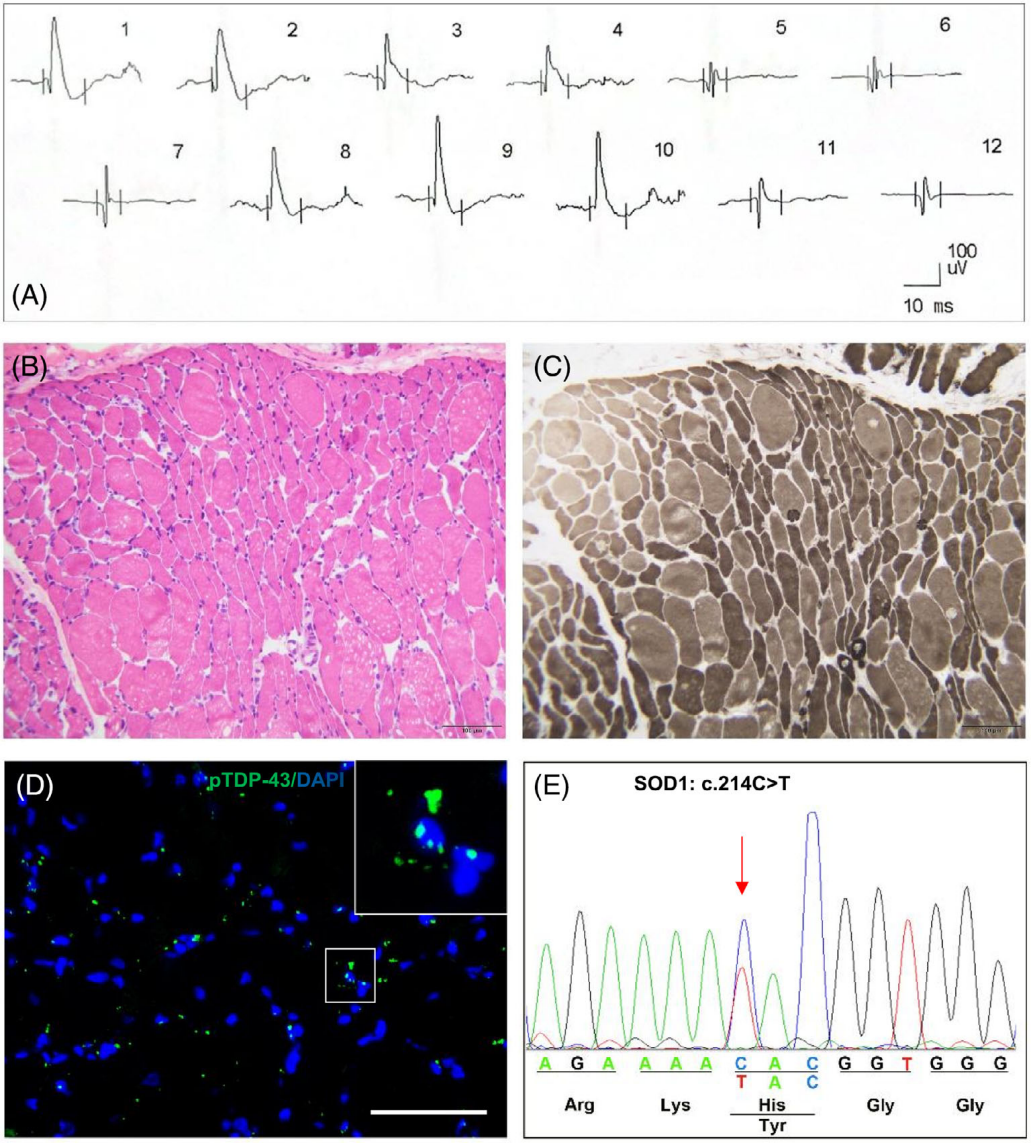
The electrophysiological feature, muscle pathology, pTDP‐43 pathology, and gene mutation in a *SOD1*‐ALS patient (ID: ALS‐05). (A) EMG studies showed short‐duration and small‐amplitude MUAPs (myopathic lesions) in left deltoideus. (B, C) After muscle biopsy, left biceps showed significant neurogenic atrophy stained with H&E and ATP PH9.6. (D) pTDP‐43 aggregates in myofibers in left biceps. (E) A pathological mutation of *SOD1* c.214C > T, p.H72Y was detected using whole‐exome sequencing. Scale bar, 100 μm (B, C); 75 μm (D).

ALS‐05 was a 52‐year‐old female who presented with progressive muscle weakness and atrophy in upper limbs and a disease duration of approximately 5 months. The clinical examinations showed LMN‐dominant lesions. EMG testing at baseline showed myopathic damage, and the duration of motor unit action potential (MUAP) was 8.3 ms (normal range: 9.36–14.04 ms; Figure 6A). However, significant neurogenic atrophy was found in the left biceps after muscle biopsy (Figure 6B,C), and pTDP‐43 had accumulated in the myofibers (Figure 6D). After whole‐exome sequencing, it was found that she carried a known mutation in the *SOD1* gene (c.214C > T, p.H72Y; HGMD: CM1515721; Figure 6E).

**FIGURE 7:**
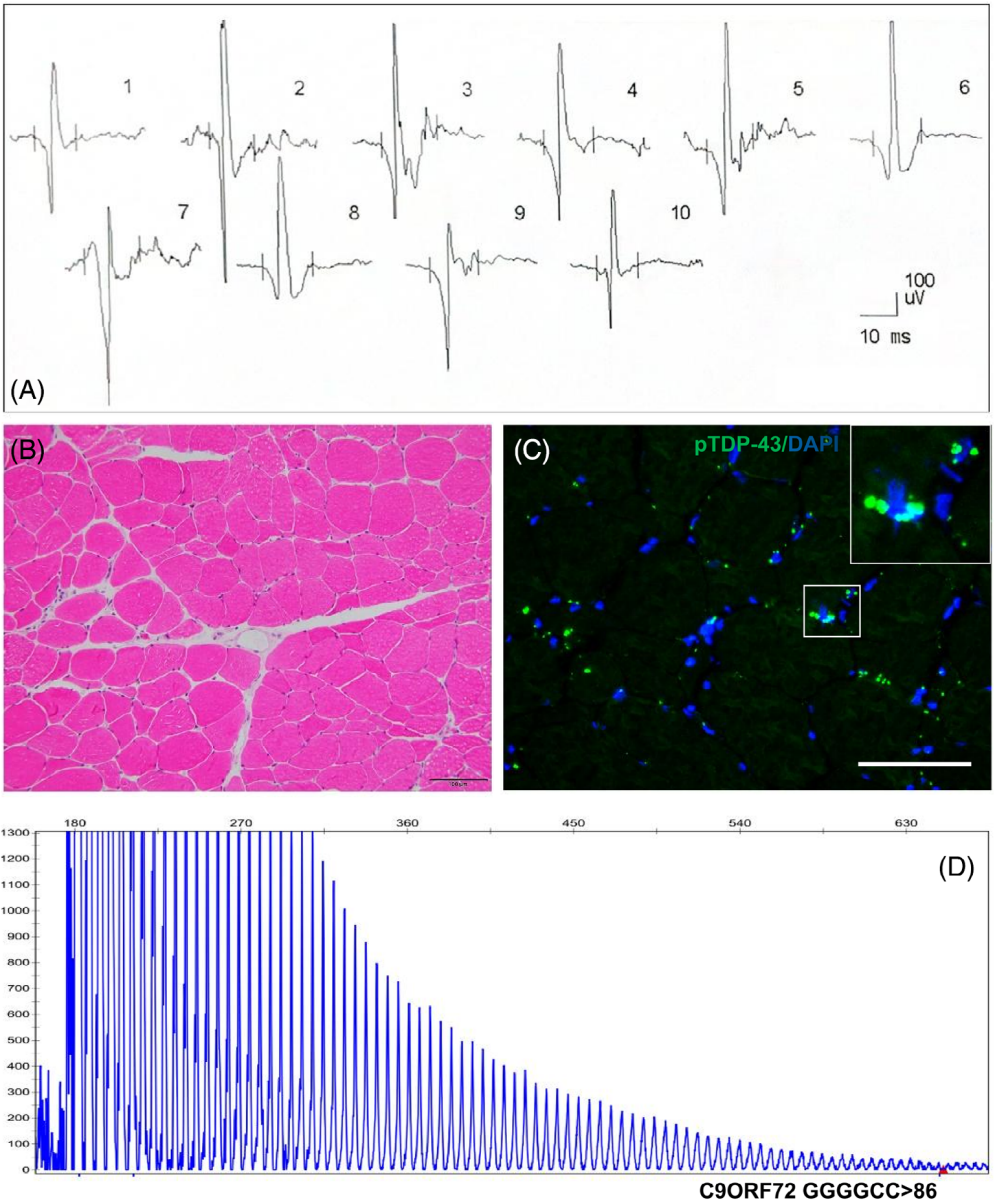
The electrophysiological feature, muscle pathology, pTDP‐43 pathology, and gene mutation in a *C9ORF72*‐ALS patient (ID: ALS‐09). (A) In EMG studies, normal‐duration of motor unit action potentials (MUAPs) in left biceps. (B) After muscle biopsy, left biceps showed mild neurogenic atrophy (H&E, arrows). (C) Obvious pTDP‐43 aggregation in myofibers in left biceps. (D) A pathological GGGGCC expansion (>86) was detected using repeat‐primed polymerase chain reaction. Scale bar, 100 μm (B); 75 μm (C).

ALS‐09 was a 57‐year‐old male patient who presented with progressive dysarthria over the course of 16 months. He had no complaints of muscle atrophy or weakness in the limbs. Typical LMN (tongue muscle weakness and atrophy) and UMN (positive jaw reflex) signs were evident. In EMG studies, the left biceps showed normal motor unit action potential (MUAP), and the duration of MUAP was 13.0 ms (normal range: 9.5–14.3 ms; Figure 7A). Only mild neurogenic atrophy was uncovered in the left biceps after muscle biopsy (H&E, Figure 7B), however, there were already several pTDP‐43 deposits (Figure 7C). He was diagnosed as having ALS with a *C9ORF72* mutation (GGGGCC repeat >86, Figure 7D).

## 4 DISCUSSION

As a neurogenetic disease center which specializes in the assessment of pathology and genetics in diverse neuromuscular diseases, we conducted this diagnostic study to investigate the value of pTDP‐43 accumulation within biopsied muscles from 72 participants, including 18 ALS patients and 54 non‐ALS controls. As anticipated, pTDP‐43 accumulation was detected in routine muscle biopsy specimens, including the biceps brachii, deltoid, tibialis anterior, and quadriceps, and accumulation was independent of muscle biopsy sites. Furthermore, through this diagnostic study, we found that the accumulation of pTDP‐43 in muscles may serve as a pathological marker for diagnosis of ALS using a semi‐quantitative pTDP‐43 score with high sensitivity (94.4%) and specificity (83.3%). In addition, for the first time we enrolled ALS patients across different King's stages and found that pTDP‐43 accumulation also occurred independently of ALS disease stage (Table S2). Among prospective patients (especially genetically confirmed ALS), it was found that muscle pTDP‐43 aggregates were present in the early stage of ALS (lacking clinical symptoms and abnormal electromyographic lesions), which confirmed pTDP‐43 aggregation as an early event in ALS and highlighted the crucial role of pTDP‐43 aggregates in ALS pathogenesis.

To date, specific pathological markers for ALS are lacking, and the diagnosis of ALS is typically based upon a combination of clinical presentation and electrophysiological testing [3]. It is difficult for clinicians to diagnose ALS in the early stage or when symptoms are atypical. Previous studies examining muscle tissue from patients at autopsy have confirmed the presence of pTDP‐43 accumulation in the cytoplasm of muscle fibers in ALS patients [10]. Kurashige et al. [11] characterized the histopathology of peripheral axons in the skeletal muscle of patients with ALS by muscle biopsy, and suggested that axonal pTDP‐43 accumulations may be characteristic of patients with ALS. However, during the same study, of the 114 patients in the muscle biopsy cohort, 37.8% did not exhibit intramuscular nerve bundles. Therefore, we selected pTDP‐43 accumulations in biopsied muscles rather than intramuscular axons as a diagnostic biomarker. In the present study, we observed that pTDP‐43 can be accumulated in routine muscle biopsy, including biceps brachii, deltoid, tibialis anterior, and quadriceps. This indicates that pTDP‐43 may function as a promising tissue marker for diagnosis of ALS.

Over 40 genetic mutations have been hitherto identified, which together account for 15% of ALS cases [3], however, the correlation between pTDP‐43 proteinopathy and genetic mutations was not fully clarified. Here, we summarized the previous studies on pTDP‐43 deposition in the muscles from ALS patients with different gene mutations, and pTDP‐43 accumulation has only been reported in *C9ORF72*‐ and *VAPB*‐ALS patients [9, 23] (Table S3). In the present study, we enrolled ALS patients with *SOD1*, *C9ORF72*, and *FUS* gene mutations, and we observed significant pTDP‐43 deposition in patients with *SOD1* and *C9ORF72* gene mutations. Previous studies were unclear about the presence of abnormal TDP‐43 accumulation in ALS patients with *SOD1* mutations. Trist et al. [24] found co‐deposition of *SOD1*, TDP‐43, and p62 proteinopathies in spinal cord motor neurons from *SOD1*‐ALS patients, while no cytoplasmic TDP‐43 immunoreactivity was observed in the prior study by Tan et al. [25] To confirm our findings in *SOD1*‐ALS patients, we invited an independent lab from us to repeat the immunostaining assay and obtained similar results (Figure S5). We provided additional evidence that pTDP‐43 aggregates were present in the muscles of *SOD1*‐ALS patients. In contrast, pTDP‐43 inclusion was absent in the ALS patient with *FUS* mutation (Figure S2), consistent with previous report in autopsy spinal cord specimens from nine ALS patients with *FUS* mutations by Nolan et al. [26] These findings indicate that pTDP‐43 has different roles in the pathogenesis of ALS with different disease‐causing gene mutations.

The pathological accumulation of pTDP‐43 in diverse neurogenic and myogenic diseases is an interesting and controversial topic. Herein, we summarized the pTDP‐43 proteinophthy in a wide range of disorders according to the previous studies (Table S4). Among the neurogenic diseases, pTDP‐43 accumulation occurs not only in ALS but also in cerebral trauma, CMT type2A2, neurolymphomatosis, vascular leukoencephalopathy, and multiple cerebral infarction [10], and our study supported the evidence of pTDP‐43 accumulation in muscle sample from NIID patient for the first time. Among the myogenic diseases, pTDP‐43 accumulation is a common pathological feature in inclusion body myositis (IBM), polymyositis, myotonic dystrophy, congenital myopathy, and mitochondrial disease [9, 10]. In this study, we added new evidence of pTDP‐43 aggregation in FSHD, GSD, LGMD, OPDM, and OPMD. Additionally, other study also reported the pTDP‐43 aggregates in autopsied muscles from myasthenia gravis (MG) patients [10]. Up to now, the origin of pTDP‐43 in muscles is controversial. A popular view is that misfolded proteins are produced by central nervous system neurons, and then spread via axons to adjacent neurons through a prion‐like mechanism [27]. However, in this study, we observed significant pTDP‐43 aggregation in the biopsied muscles of early‐stage ALS patients lacking nerve damage, which suggested that muscular pTDP‐43 deposition occurs prior to neurodegeneration in ALS. These findings highlighted the possibility that muscle tissue may be the primary site of misfolded protein production. Whether the misfolded proteins in muscles can be transmitted back to the CNS through axons under certain conditions requires further study.

As a hospital‐based center, pathology and genetics test were performed, which ensured pathological and genetic consistency for all ALS and non‐ALS patients. In addition, the large registered cohorts and use of the standardized Muscle Specimens Bank further increased the reliability of this study. However, there are several study limitations accompanying these strengths. First, we enrolled 18 ALS patients from our single medical center, and this sample size may be insufficient. Second, because of the challenging diagnosis of ALS mimics, such as other diseases affecting the lower motor neuron, and the unconventional nature of muscle biopsy, we lacked a sufficient number of ALS mimics controls. Third, a lack of appropriate platforms and technical support prevented obtaining immunoelectron micrographs of abnormal phosphorylated TDP‐43 deposition, and we hope that in future studies, we can improve the laboratory platform to fill this gap. Finally, the feasibility of pTDP‐43 as an early ALS diagnostic tool remains unclear. Despite these caveats, this study ultimately encourages further investigation. A prospective longitudinal cohort study involving a larger population with both ALS and ALS‐mimicking conditions is required moving forward.

## AUTHOR CONTRIBUTIONS

Ying Fu, Ning Wang, Zhi‐Qiang Wang, Qi‐Jie Zhang, Jie Lin, You‐Liang Wang, and Long Chen planned and designed the experiments. You‐Liang Wang, Long Chen, Ying Ding, Huan‐Huan Song, Ao‐Wei Lv, and Yu‐Ying Li performed the experiments. Qi‐Fu Guo, Wen‐Long Zhao, and Ling Fang performed clinical patient information and sample collection. Fu‐Ze Zheng, and Min‐Ting Lin organized patient muscle samples. Wei Hu, and Liu‐Qing Xu performed the electrophysiological testing. Meng‐Chao Cui prepared DANIR 8c. Zhi‐Fei Fu provided technical support for super‐resolution microscopy. You‐Liang Wang, and Long Chen performed statistical analyses of the study and interpreted the data. Jing Zhang, and Wan‐Jin Chen supervised the study. Ying Fu, Qi‐Jie Zhang, and Jie Lin wrote the manuscript and designed the figures.

## FUNDING INFORMATION

This work has been supported by the grants 82230039 (Y.F.), 82371409 (N.W.), U2005201 and U21A20360 (N.W.), from the National Natural Science Foundation of China; the grants 2022ZQNZD005 (Y.F.) from Major Scientific Research Program for Young and Middle‐aged Health Professionals of Fujian Province; the grants 2020Y9129 (Y.F.), 2021 J01209 (Q.J.Z.), and 2021Y9156 (L.F.) from the Joint Funds for the Innovation of Science and Technology of Fujian Province; the grants BPB‐LMT2021 (M.T.L.) from 2021 Provincial Special Subsidy Funds for Health (Biobank Construction Project for Neurological Diseases).

## CONFLICT OF INTEREST STATEMENT

The authors report no competing interests.

## Supporting information


Data S1.

## Data Availability

The case report form data for this study was collected within an ecosystem provided by Yidu Cloud (Beijing, China). The data that support the findings of this study are available from the corresponding author upon reasonable request via email.
