# Supplementary material for: Detection of pTDP‐43 via routine muscle biopsy: A promising diagnostic biomarker for amyotrophic lateral sclerosis
Source: Brain Pathol. 2024 Apr 11;34(6):e13261. doi: 10.1111/bpa.13261 (PMC11483524; doi:10.1111/bpa.13261)
Supplement: Supplementary file 1 — Data S1. [file BPA-34-e13261-s001.pdf]

Supplementary Table 1. The demographic and clinical characteristics of non-ALS patients

Patient ID	Gender	Age at muscle biopsy (y)	Site of muscle biopsy (1=biceps brachii ,2=deltoid,3=quadriceps,4=tibialis anterior, 5=vastus lateralis, 6= gastrocnemius, 7= soleus)	Pathological diagnosis	Mutation gene	Final diagnosis	pTDP-43 score
Ctrl-01	male	53	2	myogenic damage combined with mild neurogenic muscular atrophy	HNRNPDL	LGMD	5
Ctrl-02	male	12	3	mild myogenic damage	KCNJ2	PP	0
Ctrl-03	male	30	2	myogenic damage	GIPC1	OPDM	0
Ctrl-04	male	25	2	mild myogenic damage	FSHD1 2DRU	FSHD	0
Ctrl-05	male	31	1	myogenic damage	FSHD1 4.5DRU	FSHD	0
Ctrl-06	female	47	2	myogenic damage	FSHD1 5DRU	FSHD	1
Ctrl-07	female	20	1	mild myogenic damage	FSHD1 4DRU	FSHD	0
Ctrl-08	female	22	4	dysferlin deficient muscular dystrophy	DYSF	LGMD	0
Ctrl-09	male	20	1	mild myogenic damage	COQ7	CMT	0
Ctrl-10	female	32	5	dysferlin deficient muscular dystrophy	DYSF	LGMD	0
Ctrl-11	male	43	2	mild myogenic damage	FSHD1 5DRU	FSHD	4
Ctrl-12	male	34	1	myogenic damage	FSHD1 5DRU	FSHD	0
Ctrl-13	male	52	3	myogenic damage	HNRNPA1	LGMD	4
Ctrl-14	female	63	1	myogenic damage,dermatomyositis	ND	IM	6
Ctrl-15	female	44	2	myogenic damage	ND	IM	3
Ctrl-16	female	46	1	myogenic damage	ND	IM	0
Ctrl-17	male	68	2	myogenic damage	PABPN1	OPMD	2

Ctrl-18	male	15	2	mild myogenic damage	FSHD1 8DRU	FSHD	0
Ctrl-19	male	44	2	myogenic damage	Dystrophin	BMD	0
Ctrl-20	female	6	5	myogenic damage,dermatomyositis	ND	IM	0
Ctrl-21	male	61	2	myogenic damage,polymyositis	ND	IM	2
Ctrl-22	male	33	1	myogenic damage,mitochondrial myopathy	ND	mitochondrial disease	0
Ctrl-23	female	30	1	mild myogenic damage	GAA	GSD	0
Ctrl-24	female	18	2	mild myogenic damage	ND	mitochondrial disease	0
Ctrl-25	male	22	6	lipid storage myopathy	ETFDH	LSM	0
Ctrl-26	male	45	3	mild to moderate muscular dystrophy	HNRNPDL	LGMD	0
Ctrl-27	female	24	1	lipid storage myopathy	ETFDH	LSM	0
Ctrl-28	male	32	3	moderate muscular dystrophy	CAPN3	LGMD	0
Ctrl-29	male	41	7	mild myogenic damage	ND	LSM	0
Ctrl-30	female	67	1	oculopharyngeal muscular dystrophy	PABPN1	OPMD	3
Ctrl-31	female	58	1	myogenic damage	PABPN1	OPMD	2
Ctrl-32	female	51	6	mild myogenic damage	FSHD1 7DRU	FSHD	0
Ctrl-33	female	45	2	muscular dystrophy	DMPK	myotonic dystrophy	0
Ctrl-34	female	20	2	myogenic damage,lipid storage myopathy	ETFDH	LSM	0
Ctrl-35	female	44	2	myogenic damage,glycogen storage disease	GAA	GSD	1
Ctrl-36	female	31	3	muscular dystrophy	GAA	GSD	0
Ctrl-37	male	40	2	mild myogenic damage combined with neurogenic muscular atrophy	XK	McLeod syndrome	0

Ctrl-38	male	39	2	mild myogenic damage,lipid storage myopathy	ETFDH	LSM	0
Ctrl-39	male	20	1	myogenic damage,mitochondrial myopathy	ND	mitochondrial disease	0
Ctrl-40	male	5	1	dystrophin deficient muscular dystrophy	Dystrophin	DMD	0
Ctrl-41	male	72	4	myogenic damage combined with neurogenic muscular atrophy	NOTCH2NLC	NIID	4
Ctrl-42	male	20	3	myogenic damage	FSHD1 4DRU	FSHD	0
Ctrl-43	male	41	4	mild myogenic damage combined with neurogenic muscular atrophy	HNRNPDL	LGMD	0
Ctrl-44	male	36	3	myogenic damage	FSHD1	FSHD	0
Ctrl-45	female	50	1	mitochondrial myopathy	ND	mitochondrial disease	2
Ctrl-46	male	64	3	inflammatory myopathy	ND	IM	0
Ctrl-47	female	57	1	myotonic dystrophy	DMPK	myotonic dystrophy	0
Ctrl-48	male	27	3	normal	ND	Health (Psychogenic)	0
Ctrl-49	male	59	2	myogenic damage,inflammatory myopathy	ND	IM	5
Ctrl-50	male	12	1	myogenic damage	ND	IM	0
Ctrl-51	female	57	4	muscular dystrophy	RILPL1	OPDM	1
Ctrl-52	male	20	1	muscular dystrophy	DYSF	LGMD	0
Ctrl-53	male	69	4	rimmed vacuoles, inclusion body myositis	ND	IBM	4
Ctrl-54	female	32	1	mitochondrial myopathy	ND	mitochondrial disease	0

LGMD: limb girdle muscular dystrophy; PP: periodic paralysis; OPDM: oculopharyngodistal myopathy; FSHD: facioscapulohumeral musculardystrophy; CMT: Charcot-Marie-Tooth disease; IM: inflammatory myopathy; OPMD: oculopharyngeal muscular dystrophy; BMD: Becker muscular dystrophy; GSD: glycogen storage disease; LSM: lipid storage myopathy; DMD: Duchenn Muscular Dystrophy; NIID: neuronal intranuclear inclusion disease; IBM: inclusion body myositis; ND: not detected.

Supplementary Table 2. The muscle pTDP-43 pathological features in ALS patients with different King's College Stages based on previous studies and this study

King's College Stage	pTDP-43 pathology				reference
	Sample type	Site of sample	pTDP-43 accumulation	Proportion of pTDP43+ case	
death	autopsy	tongue, diaphragm, biceps brachii muscle,rectus femoris muscle<br>intramuscular nerve bundles	+	10/10	Kurashige,et al.[11]
NS	biopsy	intramuscular nerve bundles	+	33/33	
NS	biopsy	muscle	+	NS	Tripathi,et al.[23]
death	autopsy	axial (paraspinous, diaphragm) and appendicular muscles	+	19/57	Cykowski,et al.[9]
death	autopsy	diaphragm, iliopsoas muscle and myocardium	+	5/16	Mori,et al.[10]
		tongue, cervical muscle, diaphragm, iliopsoas muscle and myocardium	+	30/30	
1	biopsy	biceps brachii, deltoid	+	7/7	This study
2		biceps brachii, quadriceps, tibialis anterior	+	7/7	
3		biceps brachii, deltoid	+	2/3	
4		biceps brachii	+	1/1	

Supplementary Table 3. Summary of muscle pTDP-43 pathological features in ALS patients with different gene mutations based on previous studies and this study

Gene					pTDP-43 pathology				Reference
Gene	cDNA	Protein	Mutation type	Pathogenicity (ACMG)	Sample type	Site of sample	pTDP-43 accumulation	Proportion of pTDP43+ case	
C9ORF72	/	/	short tandem repeat	P	autopsy	axial (paraspinous, diaphragm) and appendicular muscles	+	4/13	Cykowski et al.[9]
VAPB	116C>T	P56S	missense mutation	P	biopsy	muscle	+	1/1	Tripathi et al.[23]
C9ORF72	/	/	short tandem repeat	P	biopsy	biceps brachii, deltoid	+	3/3	This study
SOD1	214C>T	H72Y	missense mutation	LP	biopsy	biceps brachii	+	2/2	
	449T>C	I150T	missense mutation	P	biopsy				
FUS	1574C>T	P525L	missense mutation	P	biopsy	deltoid	−	0/1	

Supplementary Table 4. The muscle pTDP-43 pathological features in non-ALS patients based on previous studies and this study

Classification of diseases	Diagnosis of pTDP43+ control	Sample type	Site of sample	Proportion of pTDP43+ case	reference
myogenic	Inclusion Body Myositis (IBM)	biopsy	axial (paraspinous, diaphragm) and appendicular muscles	4/4	Cykowski,et al.[9]
	MELAS,Mitochondrial encephalomyopathy	autopsy	diaphragm, iliopsoas muscle	1/2	Mori,et al.[10]
	myotonic dystrophy		diaphragm	2/2	
	polymyositis		cervical muscle, diaphragm, iliopsoas muscle，myocardium	1/1	
	congenital myopathy		tongue, cervical muscle, diaphragm	2/2	
	Duchenne muscular dystrophy (DMD)		myocardium	1/2	
	MERRF,Myoclonus epilepsy associated with ragged-red fibers		diaphragm	1/1	
	mitochondrial cardiomyopathy		diaphragm	1/1	
	Inclusion Body Myositis (IBM)	biopsy	tibialis anterior	1/1	This study
	inflammatory myopathy (IM)		biceps brachii, deltoid	4/8	
	mitochondrial disease		biceps brachii	1/5	
	facioscapulohumeral musculardystrophy (FSHD)		deltoid	2/10	
	glycogen storage disease (GSD)		deltoid	1/3	
	limb girdle muscular dystrophy (LGMD)		deltoid, quadriceps	2/8	
	oculopharyngodistal myopathy (OPDM)		tibialis anterior	1/2	
	oculopharyngeal muscular dystrophy (OPMD)		biceps brachii, deltoid	3/3	
neurogenic	cerebral trauma	autopsy	diaphragm, iliopsoas muscle	1/1	Mori,et al.[10]
	CMT type2A2		myocardium	1/1	
	neurolymphomatosis		tongue, cervical muscle, diaphragm, iliopsoas muscle，myocardium	1/1	
	vascular leukoencephalopathy		tongue	1/1	
	Multiple cerebral infarction		diaphragm	1/1	
	neuronal intranuclear inclusion disease (NIID)	biopsy	tibialis anterior	1/1	This study
neuromuscular junction	myasthenia gravis (MG)	autopsy	diaphragm, iliopsoas muscle，myocardium，cervical muscle	2/4	Mori,et al.[10]
other	drowning		cervical muscle, diaphragm	1/1	
	bronchopneumonia		myocardium	1/1	

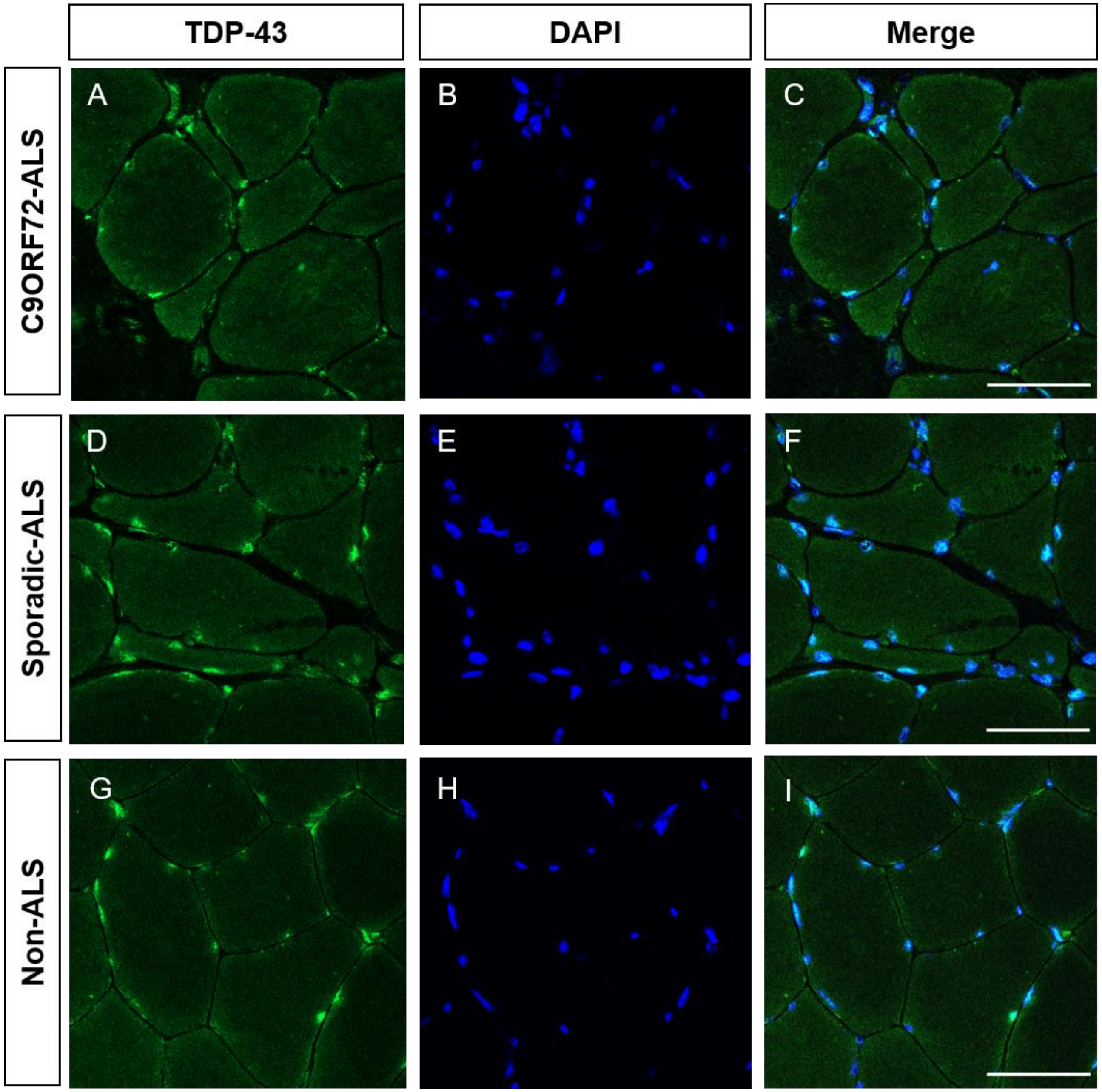

Supplementary Figure 1 Immunofluorescence stained with anti-TDP-43 in patients with C9ORF72-ALS (A-C; ID: ALS-09), sporadic-ALS (D-F; ID: ALS-12), and Health (Psychogenic) (G-I; ID: Ctrl-48). Scale bar, 50μm.

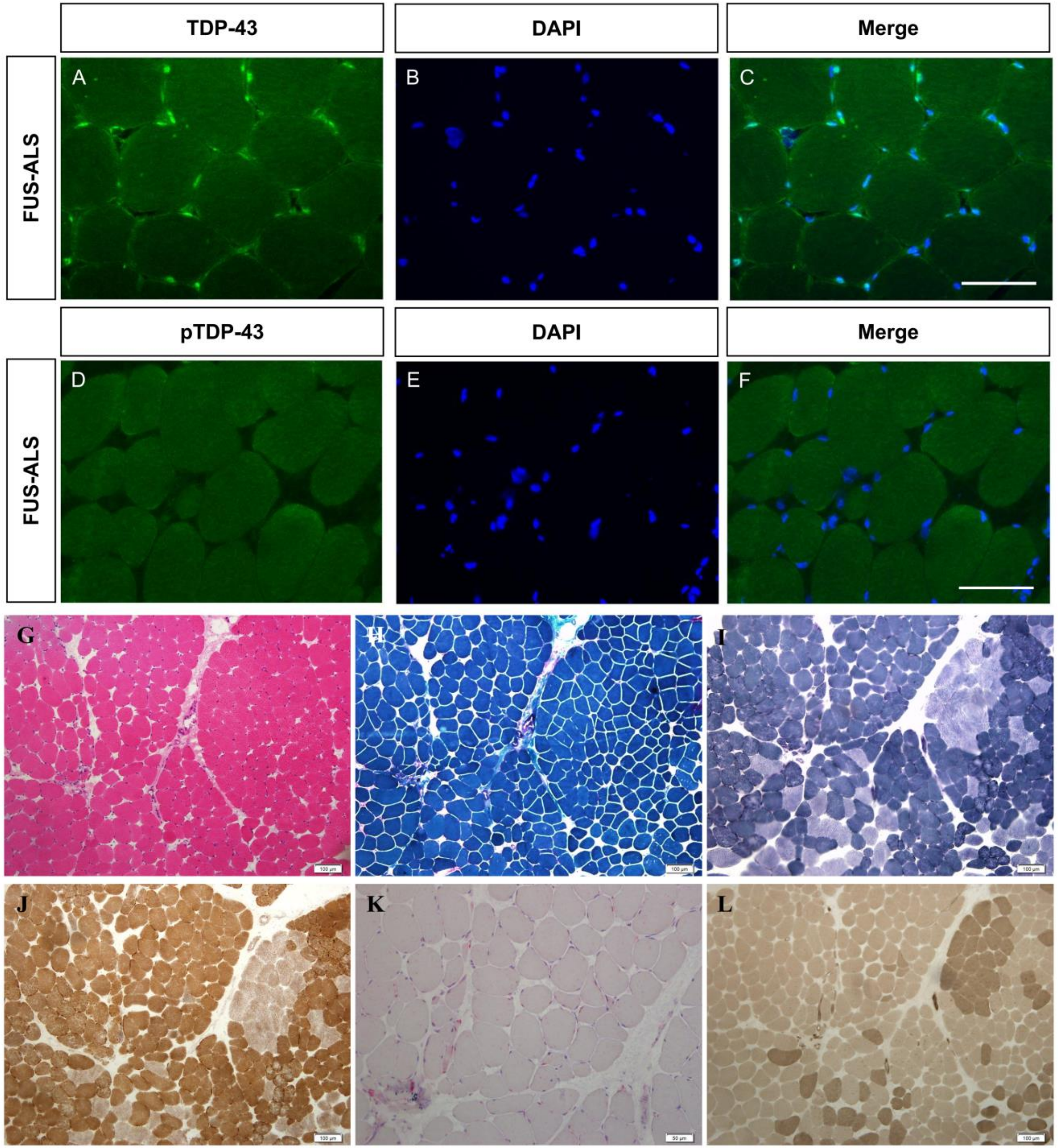
TDP-43
DAPI
Merge
FUS-ALS
A
B
C
pTDP-43
DAPI
Merge
FUS-ALS
D
E
F
G
H
I
J
K
L

Supplementary Figure 2 ALS-07 was a 16-year-old female patient, who developed progressive lower and upper limbs weakness at the age of 13. Physical examination showed UMN-dominant presentations, including hypermyotonia, brisk tendon reflexes, Hoffmann and Babinski signs. Family history was negative. She carried a de novo mutation of FUS: c.1574C>T, p.P525L. TDP-43 was expressed in the myofibers **(A-C)**, without pTDP-43 aggregation **(D-F)**. Scale bar, 75μm. Muscle pathology from the left deltoid muscle was shown. **(G)** Haematoxylin and eosin and **(H)** modified Gomori trichrome (mGT) stainings showed scattered mild to moderate atrophy of muscle fibers, mainly circular atrophy, a few hypertrophic muscle fibers, and mild hyperplasia of interstitial fiber connective tissue. **(I)** NADH-tetrazolium reductase (NADH-TR) and **(J)** succinate dehydrogenase & cytochrome C oxidase (SDH & COX) stainings showed some dark stained fibers with moth-eaten. **(K)** Acid phosphatase (ACP) staining showed no obvious abnormality. **(L)** ATPase pH 10.4 staining showed group distribution, and type I was dominant.

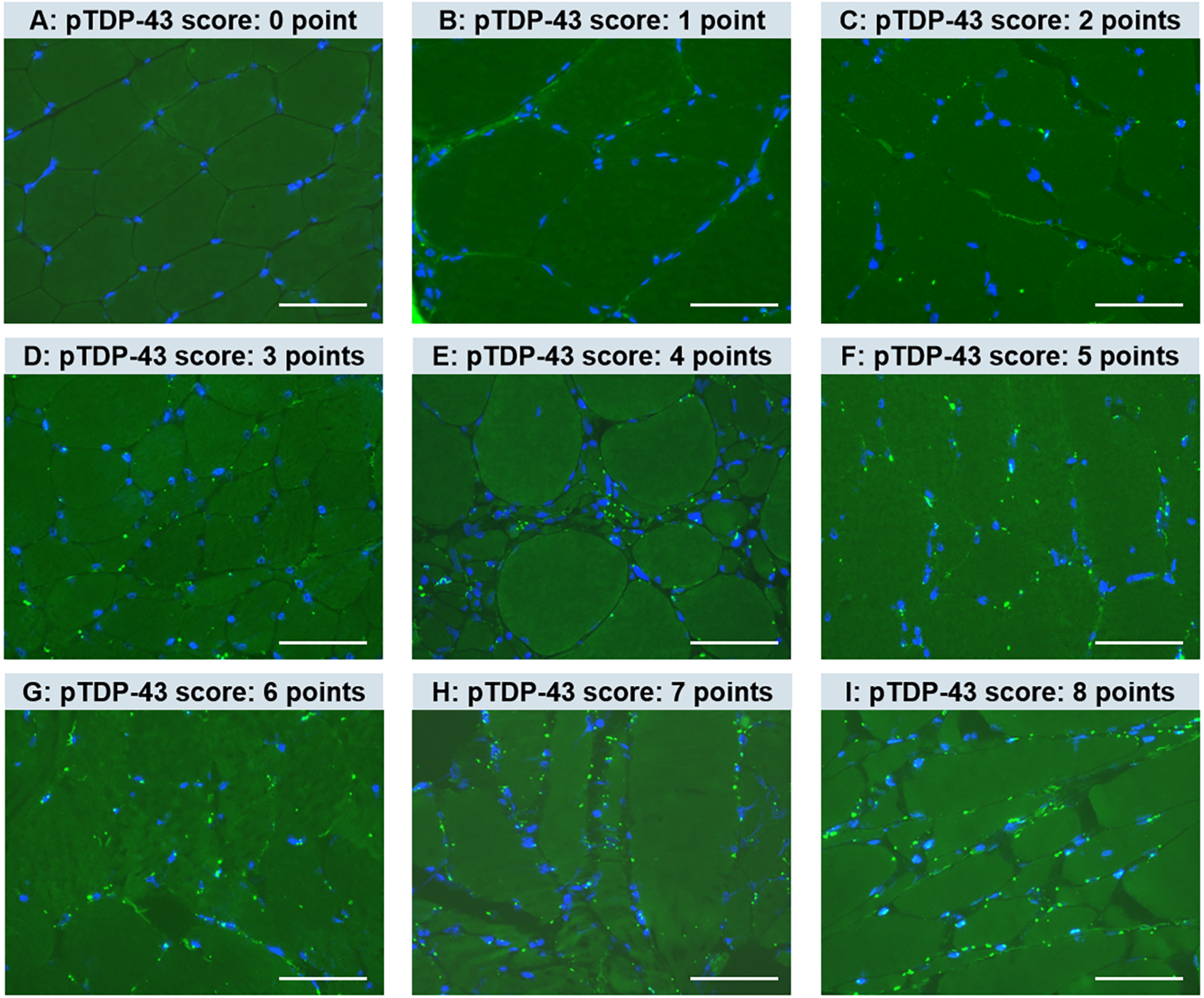

Supplementary Figure 3 The semi-quantified pTDP-43 aggregates using pTDP-43 score system. Scale bar, 75μm.

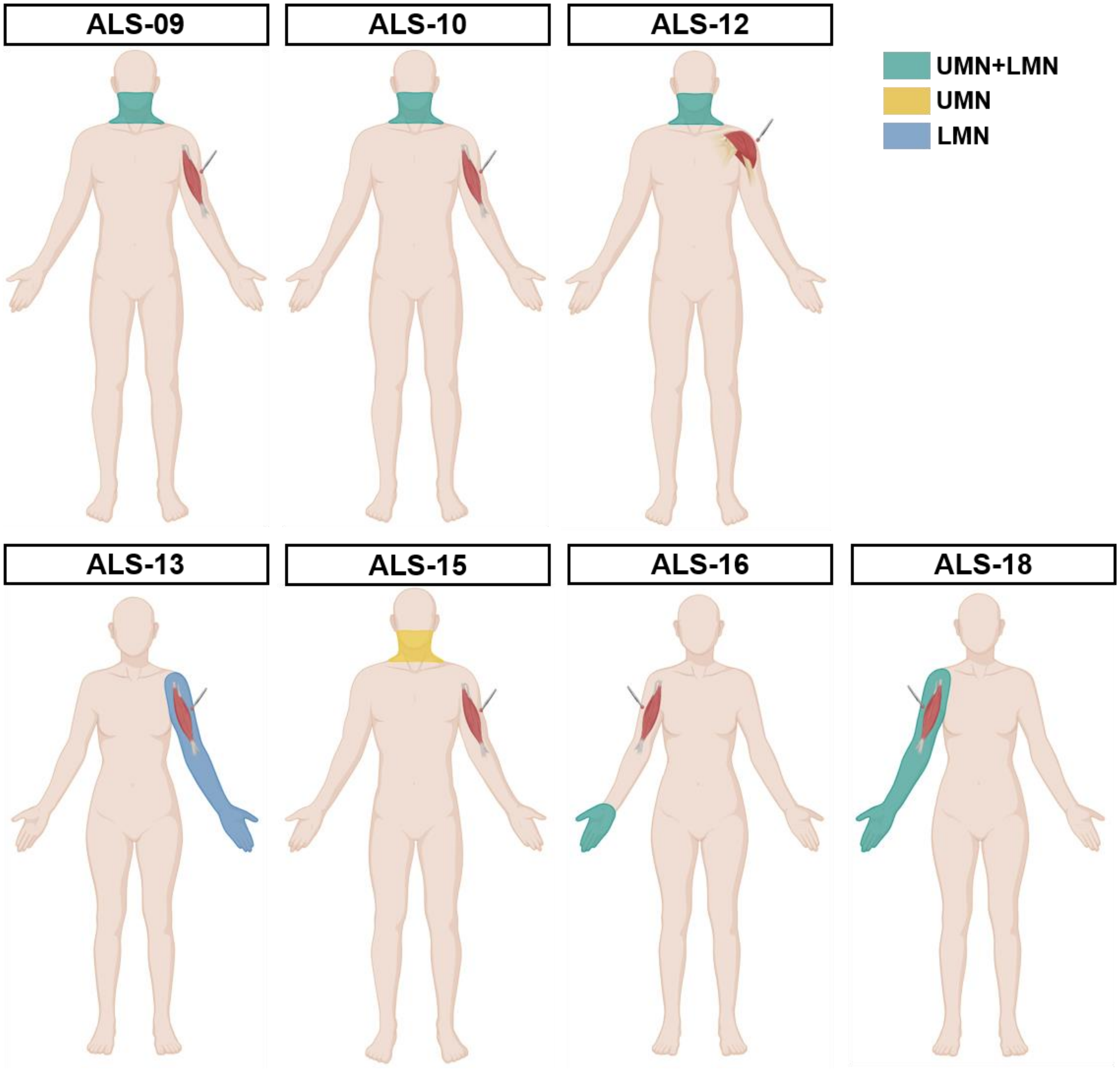
ALS-09
ALS-10
ALS-12
UMN+LMN
UMN
LMN
ALS-13
ALS-15
ALS-16
ALS-18

Supplementary Figure 4 Schematic diagram of 7 early-stage ALS patients had only 1 body region affected clinically.

ALS-09, male, with symptoms of upper and lower motor neuron damage in the bulbar (green), underwent a left biceps brachii muscle biopsy.

ALS-10, male, with symptoms of upper and lower motor neuron damage in the bulbar (green), underwent a left biceps brachii muscle biopsy.

ALS-12, male, with symptoms of upper and lower motor neuron damage in the bulbar (green), underwent a left deltoid muscle biopsy.

ALS-13, female, with symptoms of lower motor neuron damage from the left hand to the shoulder (blue), underwent a left biceps brachii muscle biopsy.

ALS-15, male, with symptoms of upper motor neuron damage in the bulbar (yellow), underwent a left biceps brachii muscle biopsy.

ALS-16, female, with symptoms of upper and lower motor neuron damage in the right hand (green), underwent a right biceps brachii muscle biopsy.

ALS-18, female, with symptoms of upper and lower motor neuron damage from the right hand to the shoulder (green), underwent a right biceps brachii muscle biopsy.UMN: upper motor neuron. LMN: lower motor neuron.

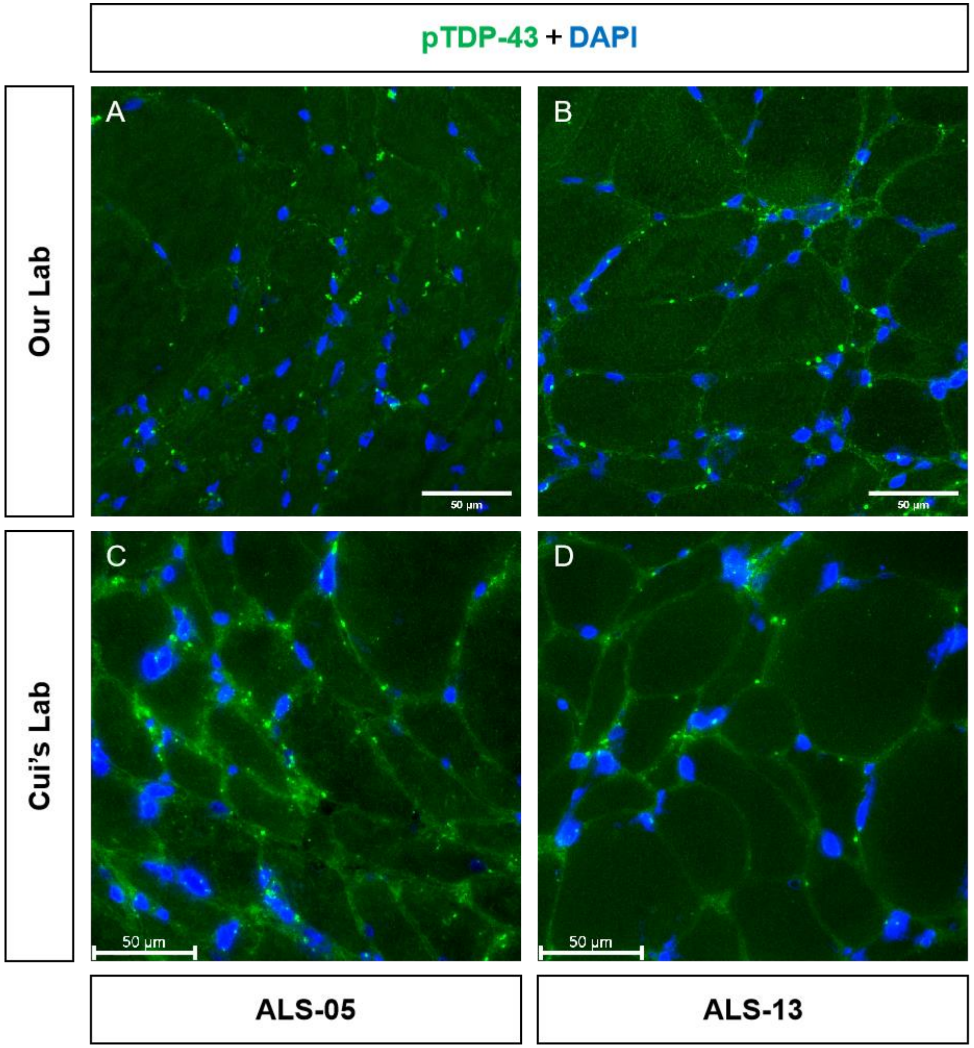

Supplementary Figure 5 Similar results about pTDP-43 immunostaining in ALS patients with SOD1 mutations which was confirmed in our laboratory and in Meng-Chao Cui's laboratory. (A-B) Immunostaining of pTDP-43 aggregation in muscle samples from 2 SOD1-ALS patients (ID: ALS-05 and ALS-13). (C-D) Immunostaining validation result of the same patients' muscle (ID: ALS-05 and ALS-13) in Meng-Chao Cui's laboratory. Scale bar, 50μm.
